# Method for isolation of high molecular weight genomic DNA from *Botryococcus* biomass

**DOI:** 10.1371/journal.pone.0301680

**Published:** 2024-07-24

**Authors:** Ivette Cornejo-Corona, Devon J. Boland, Timothy P. Devarenne

**Affiliations:** Biochemistry and Biophysics, Texas A&M University, College Station, Texas, United States of America; University of Kalyani, INDIA

## Abstract

The development of high molecular weight (HMW) genomic DNA (gDNA) extraction protocols for non-model species is essential to fully exploit long-read sequencing technologies in order to generate genome assemblies that can help answer complex questions about these organisms. Obtaining enough high-quality HMW gDNA can be challenging for these species, especially for tissues rich in polysaccharides such as biomass from species within the *Botryococcus* genus. The existing protocols based on column-based DNA extraction and biochemical lysis kits can be inefficient and may not be useful due to variations in biomass polysaccharide content. We developed an optimized protocol for the efficient extraction of HMW gDNA from *Botryococcus* biomass for use in long-read sequencing technologies. The protocol utilized an initial wash step with sorbitol to remove polysaccharides and yielded HMW gDNA concentrations up to 220 ng/μL with high purity. We then demonstrated the suitability of the HMW gDNA isolated from this protocol for long-read sequencing on the Oxford Nanopore PromethION platform for three *Botryococcus* species. Our protocol can be used as a standard for efficient HMW gDNA extraction in microalgae rich in polysaccharides and may be adapted for other challenging species.

## Introduction

The latest long-read DNA sequencing technologies have the potential to generate data to help answer genomics-based questions that were previously difficult to address due to problems producing long sequencing reads from DNA fragments of 10 kb or longer [1]. The ability to generate such sequencing data enables the assembly of genome sequences to allow researchers to categorize genes, biosynthetic pathways, and even entire organisms more accurately [2, 3]. Long-read sequencing demands high purity, high molecular weight (HMW) genomic DNA (gDNA) of ≥20 kb [4], which can be challenging for non-model species. Thus, applying these sequencing technologies to non-model organisms requires tailoring and optimizing protocols for isolation of HMW gDNA.

Microalgae are organisms that produce molecules with potential applications to many industries, making them of high scientific interest [5, 6]. However, a challenge for their industrial application is achieving optimal yields and productivity of the molecule(s) of interest in wild-type strains [7]. Genetic engineering could play a decisive role in overcoming such limitations [8, 9]. The recent availability of good quality algal genome sequences together with other omics datasets offer information to guide strategic engineering for microalgae strain improvement related to synthetic biology applications [10–12]. Particularly interesting green microalgal species are those in the genus *Botryococcus*, which can naturally accumulate acyclic hydrocarbons in the range of 30 to 60% of dry weight [13–15]. Historically, *Botryococcus* was classified as a single species, *B*. *braunii*, divided into three chemical races, A, B, and L, that were defined based on the type of hydrocarbon produced. Recently, we used genome assemblies to show that these races are actually three separate species and have been renamed *B*. *alkenealis* (formerly A race), *B*. *braunii* (formerly B race), and *B*. *lycopadienor* (formerly L race) [16].

These *Botryococcus* species are colonial and the hydrocarbons biosynthesized by these species are produced inside cells and exported into an extracellular matrix (ECM) that holds the cells of the colony together [17, 18]. The ECM is made up of cross-linked long chain aliphatic aldehydes, and the colony is surrounded by a polysaccharide sheath consisting of 2–3 μm long fibrils [18–21]. The three species of *Botryococcus* are primarily classified according to the type of hydrocarbons they produce; *B*. *alkenealis* produces C_23_-C_33_ alkadienes/alkatrienes, *B*. *braunii* produces C_30_-C_37_ triterpenes named botryococcenes, and *B*. *lycopadienor* produces the C_40_ tetraterpene called lycopadiene [21–23]. All of these hydrocarbons can be readily converted into petroleum-equivalent transportation fuels such as gasoline, jet fuel, and diesel [15]. Thus, *Botryococcus* species could be used as a source of feedstocks for the production of renewable combustion engines fuels. Despite the potential and interest in these *Botryococcus* species, the biosynthetic pathways and molecular mechanisms for hydrocarbon biosynthesis have not yet been fully described or understood, partly due to the lack of genome assemblies. This deficit has been addressed with our recent genome assemblies for *B*. *alkenealis* and *B*. *lycopadienor* [16] along with our earlier *B*. *braunii* genome assembly [24].

The *B*. *alkenealis* and *B*. *lycopadienor* genome assemblies [16] utilized a new HMW gDNA isolation protocol we developed that is optimized for *Botryococcus* biomass. The presence of high amounts of polysaccharides in the ECM of *Botryococcus* [18, 25] presents a significant challenge to obtaining a high yield of HMW gDNA, and the abundance of hydrocarbons complicates the phase separation during conventional gDNA extraction methods. Prior to developing the optimized method, we attempted to extract HMW gDNA from *Botryococcus* biomass using different commercial kits, extraction buffer formulations, and lysis conditions, but none of these approaches yielded HMW gDNA suitable for long-read sequencing. Thus, we developed a HMW gDNA extraction protocol to ensure high yield, quality, and integrity of the HMW gDNA from *Botryococcus* biomass. For example, we integrated a pretreatment step with sorbitol to remove ECM polysaccharides [26] and the brief use of sonication to properly homogenize the samples with minimal disruption of the HMW gDNA. To ensure purity, we also included PVP-40 which removes polyphenolic compounds that are often co-precipitated with nucleic acids [27], a standard RNase treatment, and a long incubation step for nucleic acid precipitation [28]. The resulting optimized protocol is an efficient method suitable for long-read sequencing of *Botryococcus* HMW gDNA [16].

## Materials and methods

The protocol described in this peer-reviewed article is published on protocols.io, https://dx.doi.org/10.17504/protocols.io.j8nlkoz2xv5r/v1, and is included for printing as S1 File with this article.

### *Botryococcus* culturing and biomass preparation

*B*. *alkenealis*, (Yamanaka strain [29]), *B*. *braunii* (Showa or Berkeley strain [30]), and *B*. *lycopadienor* (Songkla Nakarin strain [31]) were cultured in 1 L roux flasks containing 750 ml of modified Chu 13 medium pH 7.5 at 22°C under continuous aeration with filter-sterilized air enriched with 2.5% CO_2_. The cultures were maintained for 6 weeks under a 12:12 hours light:dark cycle with a light intensity of 280 μmol photons/m^2^/s. The modified Chu 13 medium contained the following chemical concentrations: KNO_3_ (0.4 g/L), MgSO_4_-7H_2_O (0.1 g/L), K_2_HPO_4_ (0.052 g/L), CaCl_2_-2H_2_O (0.054 g/L), FeNa-EDTA (0.01 g/L), H_3_BO_4_ (2.86 mg/L), MnSO_4_-H_2_O (1.54 mg/L), ZnSO_4_-7H_2_O (0.22 mg/L), CuSO_4_-5H_2_O (0.08 mg/L), NaMoO_4_-2H_2_O (0.06 mg/L) and CoSO_4_-7H_2_O (0.09 mg/L). 40-day-old fresh biomass from cultures in the stationary phase was harvested by filtration using a 10 μm nylon net, the biomass immediately frozen with liquid N_2_, and stored at -80°C.

### Buffer preparation

The sorbitol wash buffer contained 100 mM Tris-HCl pH 8.0, 0.35 M sorbitol, 5 mM EDTA pH 8.0, 1% (W/V) polyvinylpyrrolidone 40,000 MW (PVP-40), and 1% (V/V) 2-mercaptoethanol (β-ME). The DNA extraction buffer contained 100 mM Tris-HCl pH 8.0, 3 M NaCl, 3% cetyltrimethyl ammonium bromide (CTAB), 20 mM EDTA, 1% (W/V) PVP-40, and 1% (V/V) β-ME. Each of these buffers was prepared without β-ME, autoclaved, stored at 4°C, and the β-ME was added just prior to use. TE buffer contained 10mM Tris-HCl and 1mM EDTA pH 8.0. The CHCl_3_:IAA wash buffer contained a 24:1 mixture of chloroform:isoamyl alcohol and was stored at 4°C.

### DNA extraction protocol

**Fig 1:**
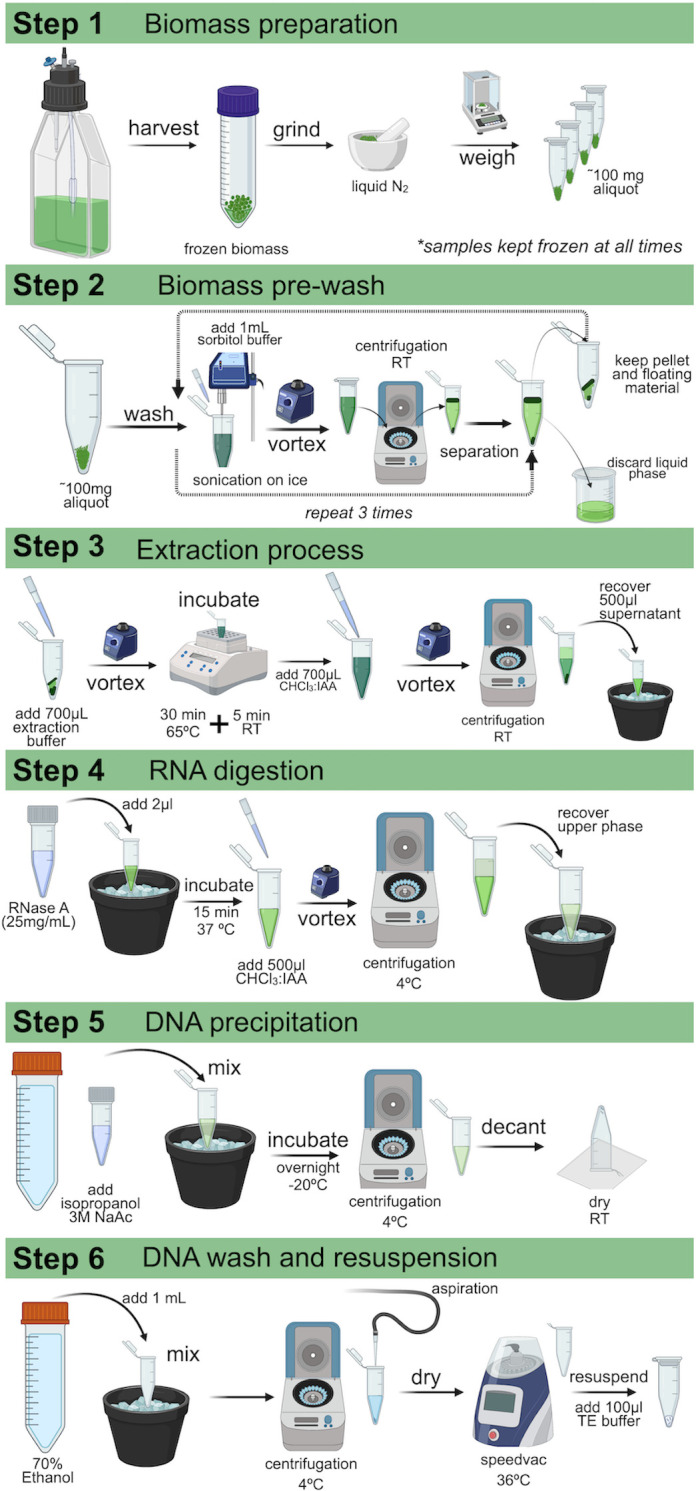
Workflow for extraction of HMW gDNA from *Botryococcus*. Created with BioRender.com.

The HMW DNA extraction protocol is summarized in Fig 1 and S1 File. Initial tests indicated optimal HMW gDNA extraction was obtained using between 100–110 mg of liquid N_2_ ground biomass. In step 1 (see Fig 1), biomass is harvested, or previously harvested biomass is removed from the -80°C freeze, the biomass ground in a mortar and pestle with liquid nitrogen, 100–110 mg samples quickly weighed to minimize thawing, samples transferred to 1.5 mL eppendorf tubes, and samples kept frozen with liquid nitrogen.

**Fig 2:**
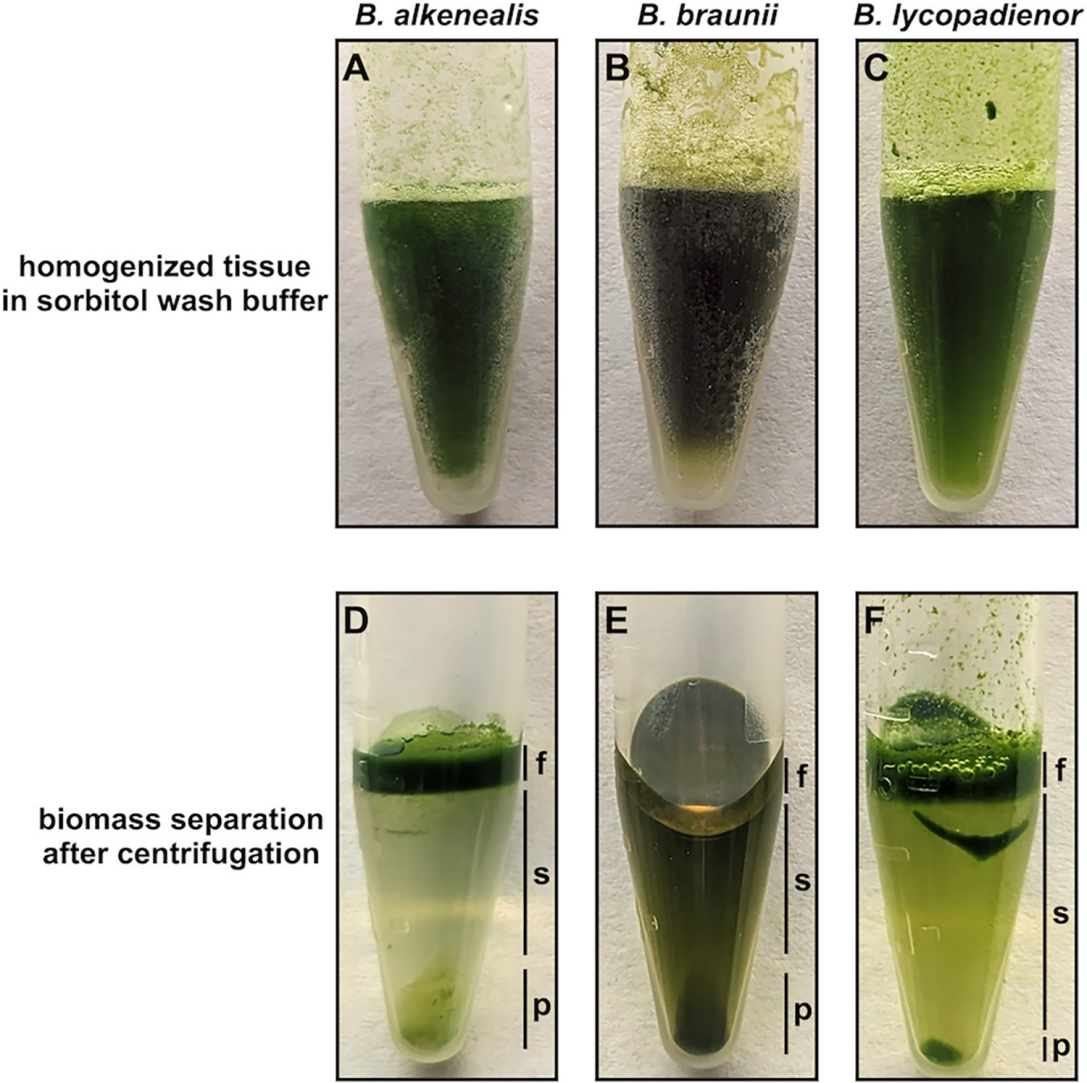
Images of ground *Botryococcus* biomass in sorbitol wash buffer before and after centrifugation. (A), (B), (C) Homogenized biomass resuspended in sorbitol wash buffer before centrifugation for each *Botryococcus* species. (D), (E), (F) Separation of biomass phases in sorbitol wash buffer after centrifugation. f, floating biomass phase; s, liquid supernatant; p, biomass pellet phase. The f and p phases are saved after each wash step.

In step 2 (see Fig 1), one ml of sorbitol wash buffer was added to each sample, the samples allowed to thaw while resuspending the biomass by vortexing (Fig 2A–2C), and samples were sonicated on ice for 25 seconds using a tip sonicator (Sonic Dismembrator, Fisher Scientific, model 100) at 30% power. Next, samples were centrifuged at 2,500 x *g* at room temperature (RT) for 5 minutes, and the liquid phase carefully removed, avoiding disruption of the floating and pelleted biomass layers formed during centrifugation (see Fig 2D–2F). The liquid phase was discarded and the sorbitol wash repeated a total of three times removing and discarding the liquid phase each time. The floating and pelleted biomass was saved and used for HMW gDNA extraction in the next step.

To start the HMW gDNA extraction in step 3 (see Fig 1), 700 μl of DNA extraction buffer (pre-warmed at 65°C) was added to each sample containing the floating and pelleted biomass, the samples resuspended by vortexing, samples incubated at 65°C for 30 minutes with mixing by inversion every 10 min, and samples incubated at RT for 5 minutes. Next, 700 μl of CHCl_3_:IAA buffer was added to each sample, samples vigorously mixed by vortexing for 10 seconds, samples centrifuged at 2,500 x *g* for 10 min at RT, and the upper aqueous phase transferred to a new 1.5 ml eppendorf tube being careful not to disturb of the debris at the phase interface. This resulted in recovery of approximately 500 μl of the aqueous phase that was then placed on ice.

In step 4 (see Fig 1), the RNA was removed by adding 2 μl of RNase A (25 mg/ml) to each sample to a final concentration of 0.1 mg/ml, the samples incubated at 37°C for 15 minutes with mixing by inversion every 5 minutes, 500 μl of CHCl_3_:IAA wash buffer was added to each sample, the samples mixed by vortexing for 10 seconds, the samples centrifuged at 13,000 x *g* for 10 minutes at 4°C, the upper phase was transferred to a fresh tube, and samples placed on ice.

In step 5 (see Fig 1), the HMW gDNA was precipitated by adding 0.1 volumes of 3M sodium acetate pH 5.2 and 0.66 volumes of cold (-20°C) isopropanol. Samples were mixed by inversion and incubated at -20°C overnight. Following incubation, samples were centrifuged at 13,000 x *g* for 10 minutes at 4°C, the supernatant removed by decanting, and tubes were left to drain by resting inverted on paper towels at RT until the samples were dry.

In step 6 (see Fig 1), the dried pellets were washed by the adding 1 ml of cold (-20°C) 70% ethanol, samples mixed by inversion, the samples centrifuged at 13,000 x *g* for 10 minutes at 4°C, the supernatants removed by aspiration trying avoid loss or disturbance of DNA pellet, and the samples dried using a vacuum centrifuge for 10 minutes at 36°C. Finally, the HMW gDNA was resuspended by adding 100 μl TE buffer, incubated for 10 minutes at RT without pipetting to minimize DNA shearing, the samples gently homogenized by inversion, and 50 μl aliquots stored at -80°C until used.

### Qualitative and quantitative analysis of isolated HMW gDNA

Approximately 200 ng of each HMW gDNA sample was analyzed by electrophoresis using a 0.5% agarose gel in Tris-acetate EDTA buffer (TAE) with 0.5 μg/ml ethidium bromide and molecular weight markers (Quick-Load 1 kb Extend DNA Ladder, NEB). Electrophoresis was performed for 15 minutes at 120 volts, and visualization was done using a UV transilluminator for a qualitative check of the abundance, size distribution, and quality for all samples. For a quantitative analysis of the HMW gDNA samples, 230 nm, 260 nm, and 280 nm absorbance readings were taken using a Biotek Epoch spectrophotometer, the DNA concentration calculated using the A_260_ reading, and A_260_/A_280_ and A_260_/A_230_ ratios calculated for assessment of protein and polysaccharide contamination, respectively.

### HMW gDNA size distribution analysis

The HMW gDNA samples from each *Botryococcus* species were sent to the sequencing facility at Cold Spring Harbor Laboratory (CSHL) for long-read sequencing. Prior to sequencing, CSHL analyzed each sample for fragment size distribution of the isolated HMW gDNA using an Agilent Femto Pulse system.

### Long-read sequencing

Long-read sequencing was carried out at the Cold Spring Harbor Laboratory (CSHL) Sequencing Technologies and Analysis core facility using the Oxford Nanopore PromethION platform. HMW gDNA libraires for each *Botryococcus* species were multiplexed on a single PromethION flow cell. Raw sequencing data was then base called and demultiplexed using Guppy v4. In total, two flow cells were run due to complications with the quality of the sequencing data on the first flow cell. The flow cell that produced lower quality data will be referred to as flow cell 1, and the flow cell with higher quality data referred to as flow cell 2. Briefly, both sets of reads from each flow cell were subjected to the same pre-assembly trimming process, however flow cell 1 had sufficiently less reads post-trim than flow cell 2, reflecting an overall lower quality of the reads obtained. The data from both flow cells were used in the final genome assemblies for *B*. *alkenealis* and *B*. *lycopadienor* [16]. However, the read summary analysis presented here (Table 1) depicts only flow cell 2 reads to present an “ideal” outcome from this method of HMW gDNA isolation. Sequencing read metrics were analyzed using a custom script (S2 File). Reads were ordered by length from largest to smallest, and standard metrics such as N50/90 BP and N50/90 NUM were determined (Table 1).

**Table 1:** Raw sequencing metrics obtained on the Oxford Nanopore PromethION platform from a single multiplexed flow cell run. Number of Reads: total number of reads obtained; Total BP: total number of base pairs sequenced; Max Read Size: size of the longest read in bp; N50 BP: total length in bp that equals 50% of the Total BP; N50 NUM: number of reads used to obtain the N50 BP value; N90 BP: total length in bp that equals 90% of the Total BP; N90 NUM: number of reads used to obtain the N90 BP value; MEAN: mean read length, MEDIAN: median read length; Coverage: ratio of Total BP to genome size.

Species	Number of Reads	Total BP	Max Read Size	N50 BP	N50 NUM	N90 BP	N90 NUM	MEAN	MEDIAN	Coverage
*B*. *alkenealis*	372,000	1,932,905,857	115,143	966,459,540	310,801	17,396,313,238	366,349	5,196	3,199	10.26x
*B*. *braunii*	540,000	2,482,531,758	111,151	741,266,543	452,388	1,334,294,241	533,571	2,745	1,709	8.71x
*B*. *lycopadienor*	468,000	1,790,448,184	140,088	895,225,222	426,850	1,611,409,780	464,001	3,826	1,880	13.2x

## Results and discussion

### Rationale for developing new HMW gDNA extraction protocol for *Botryococcus* species

In order to obtain HMW gDNA from the *Botryococcus* species *B*. *alkenealis*, *B*. *braunii*, and *B*. *lycopadienor* we optimized an extraction protocol able to remove polysaccharides while resulting in sufficient quantities of highly pure HMW gDNA of the largest sizes possible and suitable for long-read sequencing platforms. Specifically, we aimed to achieve A_260_/A_280_ and A_260_/A_230_ ratios greater than 1.8, and as much HMW gDNA as possible with fragments sizes of 50 kb or higher [6]

Extracting HMW gDNA from *Botryococcus* species has been a challenge due to the unique physical characteristics of *Botryococcus* colonies and the presence of large amounts of polysaccharides, which leads to inefficient HMW gDNA extraction [26, 32]. Since sorbitol has been established as an effective pretreatment for removing excess polysaccharides from polysaccharide-rich plant tissues [26], we used a sorbitol-based wash buffer after biomass maceration to ensure the removal of polysaccharides in our samples (Fig 1, step 2). Additionally, the CTAB-based extraction buffer used in our protocol (Fig 1, step 3) allows for the removal of any remaining polysaccharides during extraction of HMW gDNA [33–35]. As outlined below, this approach proved to be efficient in removing polysaccharides, resulted in relatively high recovery of HWM gDNA, and was suitable for long-read sequencing. However, the recovery of HMW gDNA ≥50 kb was low, especially for *B*. *braunii*.

### Overview of HMW gDNA extraction process

The HMW gDNA extraction protocol is outlined in Fig 1 and S1 File and will be briefly described here. Not all steps or details are discussed below and the Material and Methods section and S1 File should be used to obtain step by step details. Step 1 of the protocol involves harvesting, freezing, and grinding the *Botryococcus* biomass. We use a nylon mesh with a 10 μm cutoff placed over a Buchner funnel to filter the water from the biomass. We prefer the nylon mesh because the flexibility allows for easy collection of the biomass from the filter. Once filtering is complete, a rubber spatula is used to remove a small amount of biomass from the mesh, which is immediately placed in a 50 ml Falcon tube containing liquid nitrogen. This is repeated until all biomass is removed from the filter, collected into a single Falcon tube, and the Falcon tube is placed in the -80°C freezer until needed. This process allows for many small clumps of biomass to be collected into a single tube that can be easily removed for grinding without removing all the biomass. The grinding of the biomass follows standard procedures for using a mortar and pestle with liquid nitrogen. Once ground, weighing of aliquots should be done as quickly as possible to minimize thawing. Samples are transferred to eppendorf tubes and returned to liquid nitrogen.

In step 2 (Fig 1), the majority of the polysaccharides are removed with a sorbitol wash buffer that is added to the ground tissue and the samples should be homogenized in the buffer and thawed at this point (Fig 2A–2C). The polysaccharides are removed by centrifugation to separate solid biomass from the polysaccharides dissolved in the liquid phase. Since *Botryococcus* cells/colonies contain high amounts of hydrocarbons, the biomass containing hydrocarbons floats to the top of the liquid phase and biomass without hydrocarbons is pelleted (Fig 2D–2F). The liquid supernatant phase containing polysaccharides should be removed and discarded, leaving behind the floating and pelleted biomass. This process is repeated three times and after each wash the size of the pelleted biomass will increase.

In step 3, the DNA is extracted from the floating and pelleted biomass using an extraction buffer, which contains CTAB since it can remove any remaining polysaccharides and PVP-40 that removes polyphenolic compounds that are often bound to and co-precipitated with nucleic acids [33–35]. This is followed by a standard wash with chloroform:isoamyl alcohol (CHCl_3_:IAA), saving the aqueous phase for step 4.

The remaining parts of the protocol follow standard steps for DNA extractions: In step 4, the RNA in the sample is digested followed by a CHCl_3_:IAA wash, the DNA is precipitated with sodium acetate and isopropanol in step 5, and in step 6 the precipitated DNA is washed with ethanol, dried, and resuspended in TE buffer.

### Qualitative and quantitative analysis of HMW gDNA

**Fig 3:**
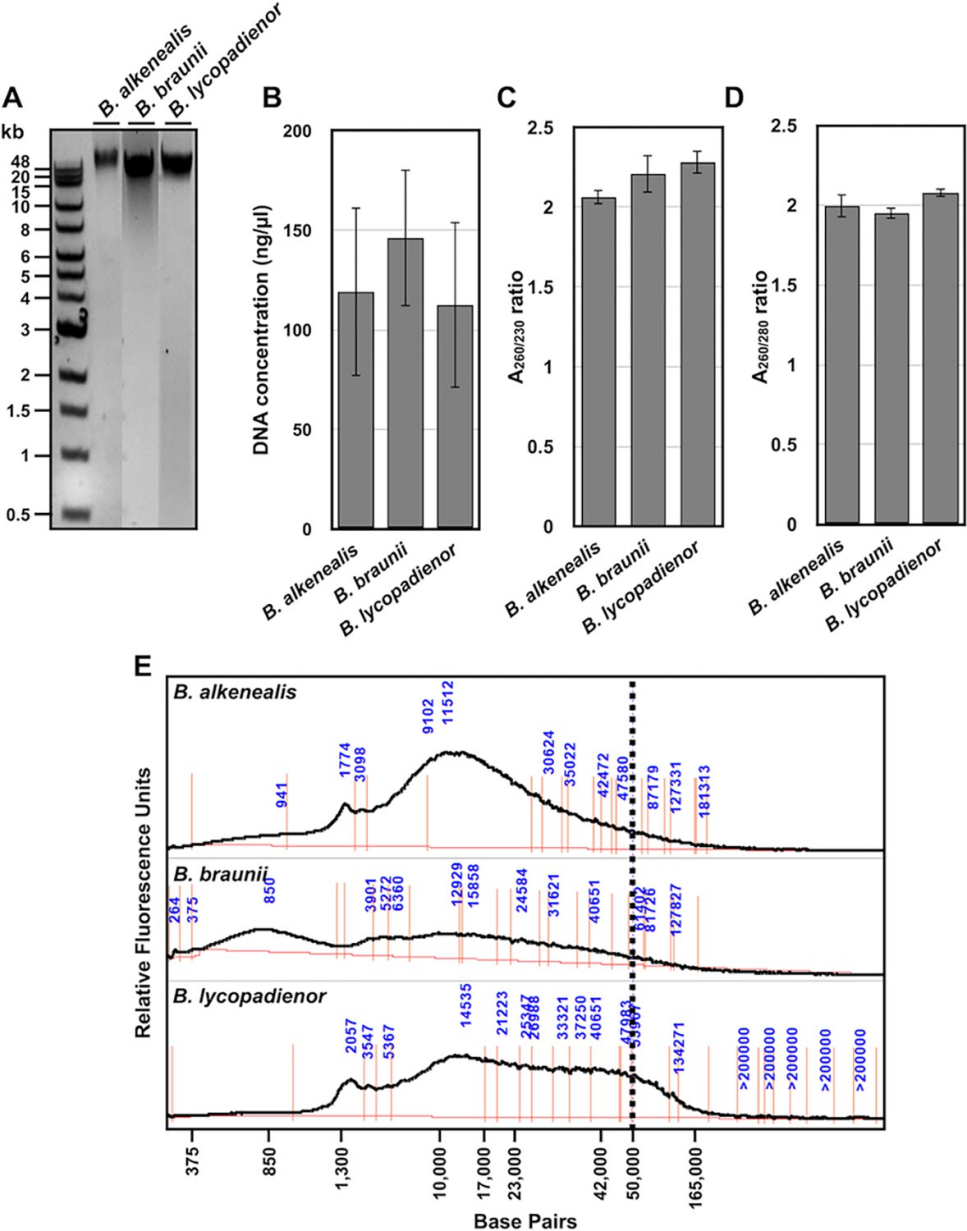
Qualitative and quantitative analysis of HMW gDNA extracted from the three *Botryococcus* species. (A) 0.5% agarose gel electrophoresis of 200 ng HMW gDNA from each *Botryococcus* species. (B) DNA concentration based on A_260_ analysis. (C) A_260/230_ ratio to assess polysaccharide contamination. (D) A_260/280_ ratio to assess protein contamination. In (C) and (D), data represents the mean ± standard error of four independent samples for *B*. *alkenealis* and *B*. *lycopadienor*, and six samples for *B*. *braunii*. A one-way ANOVA with the Friedman test was performed, and no significant differences were found between samples. (E) Size distribution of extracted HMW gDNA using an Agilent Femto Pulse system. Vertical dashed line indicates HMW gDNA ≥50kb in each sample.

The HMW gDNA isolated from each *Botryococcus* species was qualitatively analyzed by running approximately 200 ng of each sample on a 0.5% agarose gel. This analysis suggests that HMW gDNA was successfully isolated from each species with the *B*. *alkenealis* and *B*. *lycopadienor* samples having the largest molecular weight sizes of HMW gDNA followed by *B*. *braunii* (Fig 3A). The smear of lower molecular weight gDNA in the *B*. *braunii* sample (Fig 3A) may indicate enrichment of smaller gDNA fragments in this sample.

Each sample was then analyzed by absorbance spectroscopy at 260 nm (nucleic acids), 230 nm (polysaccharides), and 280 nm (protein) in order to quantitate the HMW gDNA recovery and estimate polysaccharide and protein contamination. The recovery of HMW gDNA ranged from 150 ng/μl to 225 ng/μl (Fig 3B), while the A_260_/A_280_ (Fig 3C) and an A_260_/A_230_ (Fig 3D) ratios were at 1.8 or higher for each sample indicting sufficient removal of polysaccharides and proteins. This data was obtained from four individual samples for *B*. *alkenealis* and *B*. *lycopadienor* and six samples for *B*. *braunii*. The samples for each species were pooled for the analysis in Fig 3. The raw data for each sample is shown in S1 Table.

### Size distribution of isolated HMW gDNA

The size distribution of the extracted HMW gDNA was further analyzed using the Agilent Femto Pulse System, which allows for more accurate analysis of DNA fragment size distribution as compared to agarose gel electrophoresis. The results showed that the isolated gDNA fragments in each sample spanned a large size range between 1.3 kb and 165 kb (Fig 3E). For each species the majority of gDNA fragments were in the 10 kb to 50 kb range, with *B*. *braunii* have a much lower amount of total gDNA fragments and a large amount of fragments in the 850 bp range (Fig 3E). Having a large portion of gDNA fragments of at least 10 kb in each sample (Fig 3E) is particularly important since typical long-read libraries require an insert size ranging from 500 bp to greater than 20 kb to obtain raw reads of 10 kb on average [4]. The proportion of HMW gDNA fragments recovered that were ≥50 kb was relatively small and was best for *B*. *lycopadieneor* followed by *B*. *alkenealis* and *B*. *braunii* (Fig 3E). This analysis suggests that the quality of the HMW gDNA isolated from *B*. *lycopoadienor* had the highest quality in terms of fragment length followed by *B*. *alkenealis* and *B*. *braunii* having poor quality.

### Raw sequencing metrics obtained from the isolated HMW gDNA

The purified HMW gDNA was sequenced using the Oxford Nanopore PromethION platform and was used for genome assembly of the three *Botryococcus* species. The details of how this data was processed into genome assemblies is presented in our genome analysis study for each of the *Botryococcus* species [16]. An analysis of the metrics obtained from these sequences are presented here and the data are shown in Table 1. The sequencing resulted in the generation of 372,000, 540,000, and 468,000 total sequence reads with N50s of 0.3 kb, 0.4 kb, and 0.4 kb, and a mean read length of 5.1 kb, 2.7 kb, and 3.8 kb for *B*. *alkenealis*, *B*. *braunii*, and *B*. *lycopadienor*, respectively (Table 1). Based on the predicted genome sizes for each *Botryococcus* species [24, 36], the sequence reads had genome coverages of 10.26x, 8.7x, and 13.2x, for *B*. *alkenealis*, *B*. *braunii*, and *B*. *lycopadienor*, respectively, indicating high sequencing depth. The sequences for *B*. *braunii* had the most number of sequence reads but the lowest total base pairs, mean sequence length, and genome coverage (Table 1). This suggest most of the sequence reads for *B*. *braunii* were short and supports the HMW gDNA size distribution analysis shown in Fig 3E.

## Conclusions

Developing efficient protocols for HMW gDNA extraction is indispensable to achieve good quality genome assemblies [3, 37]. Traditionally, short-read sequencing lacks the sequence length to cover complex regions within genomes such as those in plants that have large, repeat-rich genomes [38]. Long-read sequencing can overcome these problems by bridging these complex sequence regions within genomes [1]. Thus, we developed a HMW gDNA extraction protocol to isolate gDNA from *Botryococcus* species suitable for long-read sequencing.

The new HMW gDNA extraction method we developed removes the polysaccharides associated with *Botryococcus* species, resulting in HMW gDNA of high purity, in higher concentrations than previously published protocols [32], and with a large portion of the gDNA fragment sizes isolated being 10 kb or larger. Thus, this HMW gDNA was suitable for long-read sequencing platforms, and sequencing on the Oxford Nanopore PromethION platform resulted in long sequence reads suitable for genome assembly. The long-read sequences generated were used for the *de novo* genome assembly for *B*. *alkenealis* and *B*. *lycopadienor* [16]. However, the HMW gDNA and resulting long-read sequences from *B*. *braunii* were of lower quality and length, which did not allow us to improve on our previous *B*. *braunii* genome assembly [24]. It is not clear at this time why the HMW gDNA from *B*. *braunii* was of lower yield and quality as compared to *B*. *alkenealis* and *B*. *lycopadienor*. It will be interesting to see if this optimized method is suitable for the extraction of HMW gDNA from other microalgae species that have a high polysaccharide content.

## Supporting information

S1 Table Raw quantitation data for each HMW gDNA sample isolated from the three *Botryococcus* species. (PDF)

S1 File Step-by-step protocol for HMW gDNA extraction. (PDF)

S2 File PDF document with custom script for analyzing sequence reads. (PDF)
