# Supplementary material for: Method for isolation of high molecular weight genomic DNA from Botryococcus biomass
Source: PLoS One. 2024 Jul 24;19(7):e0301680. doi: 10.1371/journal.pone.0301680 (PMC11268603; doi:10.1371/journal.pone.0301680)
Supplement: S1 Table — (PDF) [file pone.0301680.s001.pdf]

Species	Sample	$A_{260/280}$	$A_{260/230}$	ng/µl	µg	Total (µg)
B. alkenealis	**1**	2.02	2.05	16.32	1.63	30.50
	**2**	1.99	2.03	254.88	25.55	
	**3**	1.89	2.05	20.40	2.04	
	**4**	2.07	2.13	16.28	1.28	
B. braunii	**1**	1.90	2.15	43.36	4.33	26.94
	**2**	1.94	2.32	37.12	3.71	
	**3**	1.92	2.28	44.00	4.40	
	**4**	1.95	2.17	50.08	5.00	
	**5**	1.80	2.06	62.80	6.28	
	**6**	2.00	2.06	32.28	3.22	
B. lycopadienor	**1**	2.07	2.28	265.88	26.58	69.28
	**2**	2.03	2.38	171.56	17.15	
	**3**	2.10	2.26	118.20	11.82	
	**4**	2.08	2.21	137.32	13.73	

S1 Table. Raw quantitation data for each HMW gDNA sample isolated from the three *Botryococcus* species. Samples for each species were pooled for long-read sequencing.
