# Supplementary material for: Method for isolation of high molecular weight genomic DNA from Botryococcus biomass
Source: PLoS One. 2024 Jul 24;19(7):e0301680. doi: 10.1371/journal.pone.0301680 (PMC11268603; doi:10.1371/journal.pone.0301680)
Supplement: S1 File — (PDF) [file pone.0301680.s002.pdf]

Description
Optimized protocol for efficient extraction of HMW gDNA from the polysaccharide-rich microalga *Botryococcus*, enabling long-read sequencing on the Oxford Nanopore Technologies platform.

Guidelines and warnings
- **Ensure Bench Cleanliness:** Prior to starting the protocol, thoroughly sanitize lab bench and instruments.
- **Maintain Frozen Samples:** It is crucial to keep samples frozen during maceration using liquid nitrogen. Prepare sterile mortar, pestle, and spatulas for this step.
- **Handle Homogenization with Care:** Exercise caution with the use of liquid nitrogen during maceration and sampling preparation.
- **Optimize Polysaccharide Removal:** For effective polysaccharide removal with minimal DNA damage, keep the sonication step prior to cell lysis brief and at a low power setting.
- **Monitor Pellet Size:** After each sorbitol wash step, check for an increase in pellet size.
- **Buffer Preparation:** Prepare and sterilize buffers in advance to streamline the process.
- **Warm DNA Extraction Buffer:** Warm the DNA extraction buffer to 65°C for at least 5 minutes before use for optimal results.

A. Buffer preparation
1. Sorbitol wash buffer. Autoclave and store this buffer at 4°C for no more than six months.
- 100 mM Tris-HCl pH 8.0
- 0.35 M Sorbitol
- 5 mM EDTA pH 8.0
- 1 % (W/V) Polyvinylpyrrolidone molecular weight 40,000 (PVP-40)
- 1% (V/V) 2-Mercaptoethanol (β-ME). Note: Add after autoclaving and before use. It is best to aliquot the amount of buffer needed and add β-ME to this aliquot.

2. DNA extraction buffer. Autoclave and store this buffer at room temperature for no more than six months.
- 100 mM Tris-HCl pH 8.0
- 3M NaCl
- 3% CTAB
- 20 mM EDTA
- 1% (W/V) Polyvinylpyrrolidone
- 1% (V/V) 2-Mercaptoethanol (β-ME). Note: Add after autoclaving and before use. It is best to aliquot the amount of buffer needed and add β-ME to this aliquot.

3. 24:1 $CHCl_3$/IAA buffer. Store this buffer at 4°C.
- 96 ml Chloroform
- 4 ml Isoamyl Alcohol.

3. 3M Sodium acetate buffer
- 408.3 g sodium acetate • $3H_2O$ per L
- pH to 5.2
- autoclave

4. 1x TE (Tris EDTA) Buffer
- 1mM EDTA, pH 8.0
- 10 mM Tris-HCl, pH 8.0

B. Culturing

***Botryococcus* culturing**

- Culture *Botryococcus* species of choice in 1 L roux flasks with 750 ml modified Chu 13 medium, pH 7.5.
- Maintain at 22°C under continuous aeration with 2.5% CO_2.
- Grow cultures for 6 weeks under a 12 h light:12 h dark cycle using 13 W compact fluorescent 65 K lighting at an intensity of 280 μmol photons/m^2/s.

C. Biomass harvesting and HMW gDNA isolation

Step 1

Biomass preparation

- Harvest the biomass by filtration using a 10 μm nylon net.
- Collect small amounts of biomass from the mesh using a rubber spatula and immediately freeze by placing in a 50 ml Falcon tube containing liquid nitrogen.
- Repeat until all biomass is collected into a single Falcom tube.
- Store at -80°C until needed.
- Place small amount of frozen biomass into mortar and pestle with liquid nitrogen.
- Grind biomass until a fine powder is formed, keeping frozen at all times.
- Weigh out ~100 mg aliquots, pace in 1.5 ml eppendorf tube, and store at -80°C.

Step 2

Biomass pre-wash

- Add 1 ml sorbitol wash buffer to 1.5 ml eppendorf tube containing ~100 mg ground biomass.
- Allow sample to thaw while vortexing for 10 seconds
- Keep samples on ice and sonicate for 25 seconds at 30% of power.
- Centrifuge at 2,500 x *g* for 5 minutes at room temperature. Discard the liquid phase by aspiration or decanting. Save the pelleted and floating biomass.
- Repeat the biomass pre-wash step three times.

Step 3

Extraction process

- Add 700 μl DNA extraction buffer pre-warmed to 65°C, homogenize by vortexing for 10 seconds.
- Incubate at 65°C for 30 minutes mixing by inversion every 10 minutes.
- Incubate samples at room temperature for 5 minutes.
- Add 700 μl $CHCl_3$:IAA buffer, vortex for 10 seconds, and centrifuge at 2,500 x *g* for 10 minutes at room temperature.
- Carefully transfer the upper aqueous phase (approximately 500 μl) to a new 1.5 ml eppendorf tube and keep on ice.

Step 4

RNA digestion

- Add 2 μl RNase A (25 mg/ml), and incubate at 37°C for 15 minutes mixing by inversion every 5 minutes.
- Add 500 μl $CHCl_3$:IAA buffer, vortex 5 seconds, and centrifuge at 13,000 x *g* for 10 minutes at 4°C.
- Transfer the upper phase to a new 1.5 ml eppendorf tube and keep on ice.

Step 5

HMW gDNA precipitation

- Precipitate the HMW gDNA by adding 0.1 volumes of 3M sodium acetate pH 5.2 and 0.66 volumes of cold (-20°C) isopropanol.

- Incubate samples overnight at -20°C.
- Centrifuge at 13,000 x *g* for 10 minutes at 4°C, and discard supernatant by aspiration or decanting.
- Dry pellet by resting inverted on paper towels at room temperature.

Step 6

HMW gDNA wash and resuspension

- Wash dried pellets with 1 ml of 70% ethanol and invert several times.
- Centrifuge at 13,000 x *g* for 10 min at 4°C. Remove the supernatant by aspiration to avoid pellet disturbance.
- Dry samples in a vacuum centrifuge for 10 min at 36°C.
- Resuspend HMW gDNA by adding 100 µl 1x TE buffer. Incubate at room temperature for 10 min then gently homogenize by inversion. Avoid pipetting that will shear the DNA. Store at -80°C until needed.

D. Qualitative and quantitative analysis of HMW gDNA

1. Qualitative analysis.

- Analyze 200 ng of HMW gDNA by electrophoresis using a 0.5% agarose gel in 1x TAE buffer.
- Carry out electrophoresis for 15 min at 120 volts and visualize with UV transilluminator.

2**. Quantitative analysis.**

- Measure absorbance at 230 nm (polysaccharides), 260 nm (nucleotides), and 280 nm (protein) using a UV-VIS spectrophotometer.
- Calculate DNA concentration using the A_{260} reading.
- Calculate the A_{260}/A_{280} and A_{260}/A_{230} ratios to estimate protein and polysaccharide contaminations, respectively. Ratio values of 1.8 or higher are preferred.
