# Supplementary material for: Method for isolation of high molecular weight genomic DNA from Botryococcus biomass
Source: PLoS One. 2024 Jul 24;19(7):e0301680. doi: 10.1371/journal.pone.0301680 (PMC11268603; doi:10.1371/journal.pone.0301680)
Supplement: S2 File — (PDF) [file pone.0301680.s003.pdf]

```
"""
Purpose: To take a fastq.gz file of reads (tested with Nanopore data)
and perform basic quality metric analysis
such as N50/N90, mean, and median calculations

This is a fully programmed script, that can be run in an enviromenmt
with python3, argparse, and BioPython modules installed.
To use, simply run python3 path/to/script.py

Created By: Devon J. Boland
"""

from Bio import SeqIO
import argparse as ap
import os


####### ARGUMENT BLOCK FOR PROGRAM #########
parser = ap.ArgumentParser(
    prog="Sequencing & Length Ultra Reading Machine (SLURM)",
    description="A \"seemingly\" lightweight python program designed
to compute summary statistics for sequencing read data. \
        Originally deisgned for Oxford Nanopore Sequencing data but
tested for Illumina and PacBio data well.",
    epilog="Be on the lookout for new Diet SLURM and return of Classic
SLURM"
)

parser.add_argument("-f", "--file", type=str, help="Path to file for
analysis")
parser.add_argument("--file_type", type=str, help="Format of
sequencing data file fasta or fastq. (default: fastq)")

args = parser.parse_args()

try:
    args.file_type == "fasta" or "fastq"
except:
    print("--file_type requires fasta or fastq passed as an argument,
other formats are not supported at this time.")
    quit()


if args.file_type == "fasta":
    from Bio.SeqIO.FastaIO import SimpleFastaParser as iterator

elif args.file_type == "fastq":
    from Bio.SeqIO.QualityIO import FastqGeneralIterator as iterator

######### END ARGUMENT BLOCK ###############
```

```
############ Define Sort Reads Function ##########

def sort_function(file):
    len_and_ids = sorted((len(rec), rec.id) for rec in
SeqIO.parse(file, args.file_type))
    ids = [id for (length, id) in len_and_ids]
    del len_and_ids
    record_index = SeqIO.index(file, args.file_type)
    records = (record_index[id] for id in ids)
    SeqIO.write(records, "sorted.fasta", args.file_type)
    print("Data is Sorted!")
    return ids[-1]

############ End Sort Reads Function #######

####### Define Metric Function #############
def get_n_metric(cutoff):
    bp= 0
    num= 0
    count = 0
    length = 0
    with open("sorted.fasta", "r") as to_caluclate:
        if args.file_type == "fasta":
            for title, seq in iterator(to_caluclate):
                count += 1
                length += len(seq)
                if length >= cutoff:
                    bp = length
                    num = count
                    break
            return bp, num
        elif args.file_type == "fastq":
            for title, seq, qual in iterator(to_caluclate):
                count += 1
                length += len(seq)
                if length >= cutoff:
                    bp = length
                    num = count
                    break
            return bp, num
########### End Metric Function ##############


###### Define Calculate Metrics Function #####

def calculate_metrics(sorted_data,last_id):
    seq_count = 0
    total_seq_length = 0
    with open(sorted_data, "r") as to_analyze:
```

```
        if args.file_type == "fasta":
            for title, seq in iterator(to_analyze):
                seq_count += 1
                total_seq_length += len(seq)
            Median_contig = round((seq_count+1)/2)
            to_get_max = SeqIO.index(sorted_data, args.file_type)
            max_value = len(to_get_max[last_id])
            to_get_median = list(SeqIO.parse(sorted_data, 
args.file_type))
            median_value = len(to_get_median[Median_contig])
            Mean = round(total_seq_length/seq_count)
            N90 = round(total_seq_length*0.9)
            N50 = round(total_seq_length*0.5)
        elif args.file_type == "fastq":
            for title, seq, qual in iterator(to_analyze):
                seq_count += 1
                total_seq_length += len(seq)
            Median_contig = round((seq_count+1)/2)
            to_get_max = SeqIO.index(sorted_data, args.file_type)
            max_value = len(to_get_max[last_id])
            to_get_median = list(SeqIO.parse(sorted_data, 
args.file_type))
            median_value = len(to_get_median[Median_contig])
            Mean = round(total_seq_length/seq_count)
            N90 = round(total_seq_length*0.9)
            N50 = round(total_seq_length*0.5)
        N50_bp, N50_num = get_n_metric(N50)
        N90_bp, N90_num = get_n_metric(N90)
        summary_dict = {"NUM": seq_count, "MAX": max_value, "N50 BP": 
N50_bp, "N50 NUM": N50_num, "N90 BP": N90_bp, \
                            "N90 NUM": N90_num, "MEAN": Mean, 
"Median": median_value, "BP": total_seq_length}

    for group in summary_dict:
            print(group, summary_dict[group])
    with open('summary.csv', 'a+') as f:
        filename = os.path.basename(args.file)
        base_only = filename[:filename.rindex('.')]
        f.write("%s\n"%(base_only))
        for key in summary_dict.keys():
            f.write("%s,%s\n"%(key,summary_dict[key]))
    return

####### End Calculate Metrics Function #########

to_search = sort_function(args.file)
calculate_metrics("sorted.fasta", to_search)
print("Cleaning up intermediate files........")
os.remove("sorted.fasta")
print("All done")
```
